# Optimized-Unet: Novel Algorithm for Parapapillary Atrophy Segmentation

**DOI:** 10.3389/fnins.2021.758887

**Published:** 2021-10-13

**Authors:** Cheng Wan, Jiasheng Wu, Han Li, Zhipeng Yan, Chenghu Wang, Qin Jiang, Guofan Cao, Yanwu Xu, Weihua Yang

**Affiliations:** ^1^College of Electronic and Information Engineering/College of Integrated Circuits, Nanjing University of Aeronautics and Astronautics, Nanjing, China; ^2^School of Electronic Information and Communications, Huazhong University of Science and Technology, Wuhan, China; ^3^The Affiliated Eye Hospital of Nanjing Medical University, Nanjing, China; ^4^Intelligent Healthcare Unit, Baidu, Beijing, China

**Keywords:** medical image segmentation, high myopia, parapapillary atrophy, convolutional neural network, fundus image

## Abstract

In recent years, an increasing number of people have myopia in China, especially the younger generation. Common myopia may develop into high myopia. High myopia causes visual impairment and blindness. Parapapillary atrophy (PPA) is a typical retinal pathology related to high myopia, which is also a basic clue for diagnosing high myopia. Therefore, accurate segmentation of the PPA is essential for high myopia diagnosis and treatment. In this study, we propose an optimized Unet (OT-Unet) to solve this important task. OT-Unet uses one of the pre-trained models: Visual Geometry Group (VGG), ResNet, and Res2Net, as a backbone and is combined with edge attention, parallel partial decoder, and reverse attention modules to improve the segmentation accuracy. In general, using the pre-trained models can improve the accuracy with fewer samples. The edge attention module extracts contour information, the parallel partial decoder module combines the multi-scale features, and the reverse attention module integrates high- and low-level features. We also propose an augmented loss function to increase the weight of complex pixels to enable the network to segment more complex lesion areas. Based on a dataset containing 360 images (Including 26 pictures provided by PALM), the proposed OT-Unet achieves a high AUC (Area Under Curve) of 0.9235, indicating a significant improvement over the original Unet (0.7917).

## Introduction

Myopia is a common eye disease, which refers to the blur of vision when light enters the eye and gathers in front of the human retina (Saw et al., 1996). Some patients experience symptoms such as headache and eye fatigue. Myopia is the main cause of vision loss worldwide (Fredrick, 2002). Vision can be corrected using glasses, contact lenses, and refractive surgery; however, they do not solve the potential defects (Morgan et al., 2012; Dolgin, 2015). In recent years, the global proportion of patients with myopia has been increasing (Holden et al., 2016). Adolescent myopia has become a common phenomenon in East and Southeast Asia (Morgan et al., 2018). The degree of myopia is usually divided by the size of the diopter D, which is divided into mild myopia (−3 D or below), moderate myopia (−3 D to −6 D), and high myopia (−6 D or above) (Saw et al., 2005). Patients with high myopia are more likely to have retinal detachment, and the probability of suffering from glaucoma is higher. Floating objects and shadows appear in the field of vision in many highly myopic patients. The medical burden of high myopia includes pathological complications such as myopic macular degeneration, choroidal neovascularization, cataracts, and glaucoma (Pan et al., 2012).

Parapapillary atrophy (PPA) often occurs in the fundus of patients with high myopia. The extent and development of PPA are useful medical assessment tools because they are closely related to the severity of several eye diseases and conditions, including glaucomatous optic nerve damage, visual field defects, and myopia (Heijl and Samander, 1985; Park et al., 1996; Uchida et al., 1998; Dai et al., 2013). The size and position of PPA are not fixed. If it progresses to the macular area, patients will find it difficult to see objects close to them. Generally, it is determined whether it is still expanding according to the shrinking edge. A clear edge indicates that the PPA has probably stopped progressing; the fuzzy and irregular edges indicate that it is still progressing.

Currently, convolutional neural networks are widely used in the field of medical diagnosis (Fang et al., 2020; Xia et al., 2020; Yang et al., 2021). This study attempts to use a new type of convolutional neural network (OT-Unet) to automatically segment PPA. The OT-Unet is based on Unet, using three pre-training models: VGG, ResNet, and Res2Net in the convolutional feature extraction stage. The edge attention, parallel partial decoder, and reverse attention modules were added to the network, and the loss function was improved simultaneously. Considering these improvements, the accuracy of the network segmentation has significantly improved.

## Materials and Methods

### Data Acquisition and Processing

**FIGURE 1:**
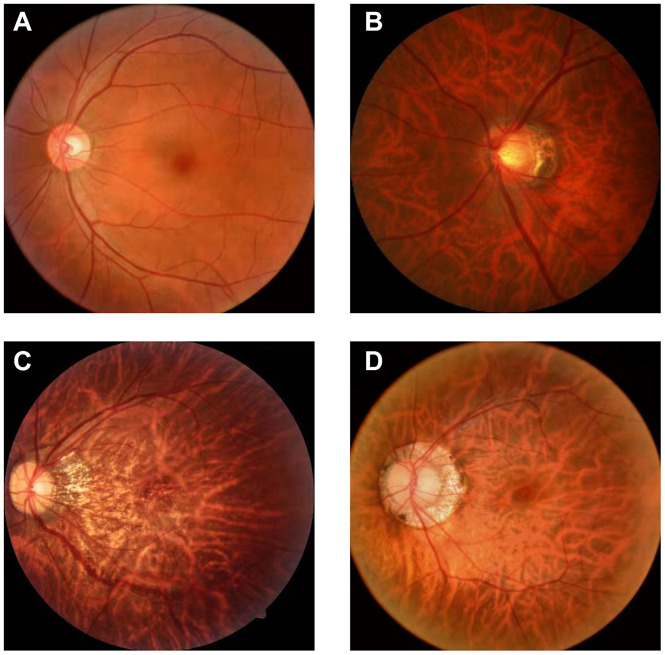
Color photo of fundus: **(A)** fundus of normal eye: It has clear boundary and clear blood vessels, and the overall color is rosy; **(B)** fundus of high myopia: It has blurred boundary, parapapillary atrophy and leopard eye fundus; **(C)** progressive parapapillary atrophy: It has blurred boundary; **(D)** unprogressive parapapillary atrophy: It has clear borders.

**FIGURE 2:**
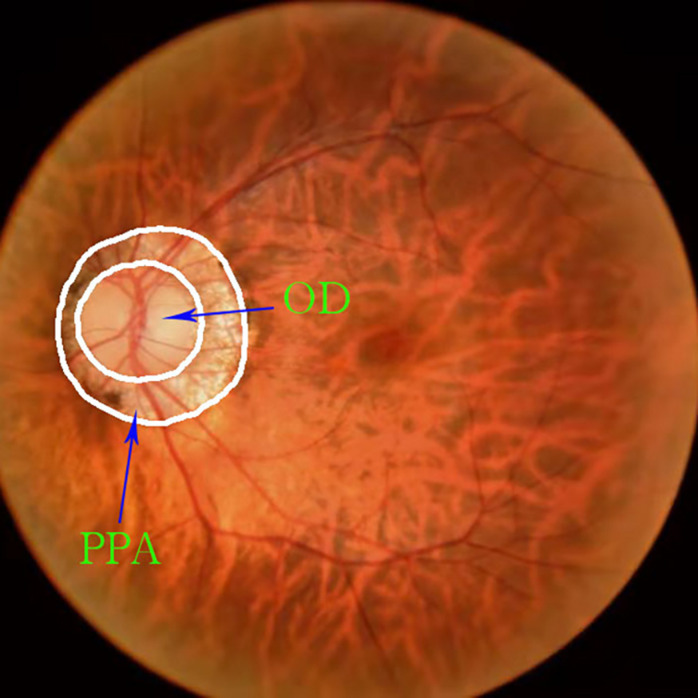
Relationship between the position of the parapapillary atrophy and optic disc: The part inside the white circle in the middle is optic disc, and the ring part between the two white circles is parapapillary atrophy.

There are few datasets of color fundus photos for high myopia, and only 26 images on iChallenge-PALM can be retrieved on the Internet. This cannot meet the needs of segmentation network training. The research team obtained more than 400 datasets from the Affiliated Eye Hospital of Nanjing Medical University. The shooting equipment was a Topcon TRC-NW300 non-mydriatic fundus camera. Preliminary processing of the data was performed. The blurred pictures and pictures with severely deformed fundus were removed, the rectangular pictures were cut into squares, and the size was unified. Finally, 360 color fundus photos of better quality were obtained. Figure 1 shows color photo of fundus. The labelme tool was used to label the PPA, and the labeling was performed under the guidance of a professional doctor. Figure 2 shows the relationship between the position of the parapapillary atrophy and optic disc.

**FIGURE 3:**
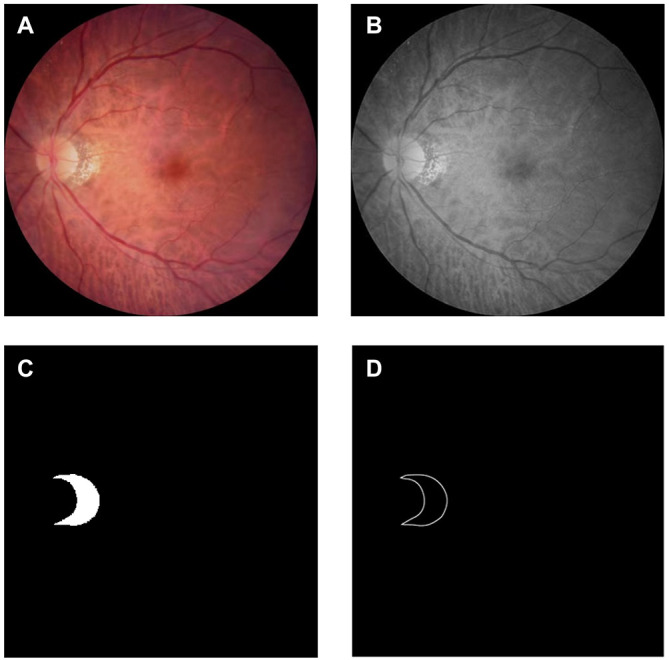
Dataset: **(A)** color photo of the fundus of high myopia: Obtained using Topcon TRC-NW300 non-mydriatic fundus camera; **(B)** grayscale image of the fundus of high myopia: Apply Adobe PhotoShop 2019 to grayscale images; **(C)** gorund truth mask of the parapapillary atrophy: Obtained by labeling the grayscale image using labelme; **(D)** edge map of the parapapillary atrophy: Obtained by processing the real label image using Adobe Illustrator 2019.

The resolution of the color fundus photos used in the experiment was 352 × 352, and the edge truth map was obtained from the real label map using Adobe Illustrator 2019. The dataset was certified by a professional doctor. According to the ratio of 4:1, we divided the data set into 288 training pictures and 72 test pictures. To highlight the PPA while reducing the size of the image, the grayscale fundus photos were obtained. This operation was implemented using Adobe PhotoShop 2019. Figure 3 shows the original image and its corresponding grayscale map, PPA truth map and PPA edge map.

### Optimized-Unet Overview

**FIGURE 4:**
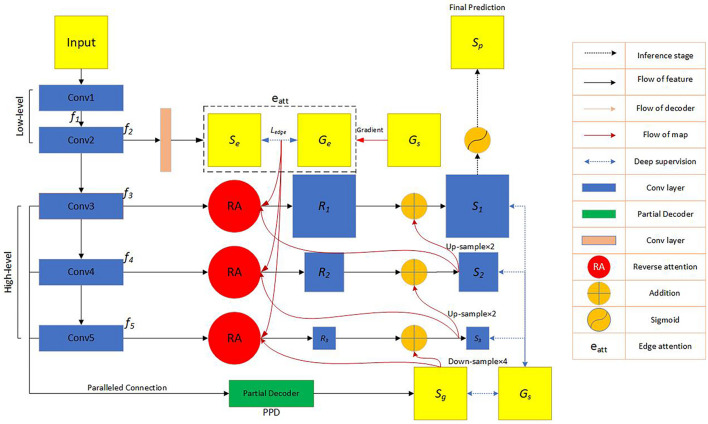
Flow chart of the automatic network segmentation: In the convolutional feature extraction stage, three pre-training models of VGG, ResNet and Res2Net are used, respectively, and the edge attention module, parallel partial decoder module and reverse attention module are added to enhance the feature expression ability. The model also improves the loss function. *f_i* represents the feature information obtained by the image through the *i*-th convolutional layer. *S*_*e*_ represents the feature information generated by the second convolutional layer information through the edge attention module. *G_e* represents the edge map obtained by the ground truth map.*L*_*edge*_ represents the standard binary cross-entropy loss function. *G_s* represents the ground truth map. *S_g* represents the feature information obtained by the parallel partial decoder aggregating high-level convolutional layer information. *R*_*i*_(i = 1,2,3), respectively, represent the feature information output by the three reverse attention modules. *S*_*i*_(i = 1,2,3), respectively, represent the information obtained by the combination of the reverse attention module information and the higher-level information.

The network block diagram of the OT-Unet algorithm proposed in this study is shown in Figure 4. The network uses Unet as the backbone network and introduces a pre-training model to generate five convolutional layers. The first two layers {*f*_*i*_,*i* = 1,2} are used to extract low-level feature maps that are rich in contextual information, and the high-level feature maps extracted from the last three layers {*f*_*i*_,*i* = 3,4,5} include more local information. An edge attention module is added between the low- and high-level feature convolutional layers to extract the edge feature information of the lesion area. Using the edge feature enhancement module in the second convolutional layer can extract richer local information. Compared with the first convolutional layer, the resolution of the image is lower, which can speed up the calculation. Simultaneously, parallel partial decoders are used to aggregate multi-scale high-level feature information to generate a global map. Since the aggregation of low-level features does not significantly improve the segmentation performance of the network, the network chooses to aggregate three high-level features to obtain richer joint feature information. In addition, the low-level feature map is input to the reverse attention module at all levels under the effect of the global map. These reverse attention modules are cascaded with each other to aggregate the low- and high-level feature information. It can be seen from the figure that the second convolutional layer information, the high-level convolutional layer information, and the aggregated information output by the parallel partial decoder are connected and processed in the reverse attention module. In addition, the use of three reverse attention modules ensures that the network generates sufficiently rich aggregate information. The feature information generated by the last-level inverse attention module is activated by the sigmoid activation function to generate the final lesion area segmentation prediction map. The following will be introduced in detail: the backbone network (Unet), key modules, and improved loss functions.

### Backbone Network—Unet

In 2015, Ronneberger et al. (2015) proposed the Unet structure. Unet is a semantic segmentation network based on Fully Convolutional Networks (FCN), which is currently widely used in the field of biomedical image segmentation. The segmentation network system includes contraction (also known as an encoder) and expansion (also known a decoder) paths.

### ImageNet’s Pre-trained Model

#### Visual Geometry Group

In 2014, Simonyan and Zisserman (2014) proposed a new network known as the VGG. The image passes through the convolutional layer, and the filter uses a very small receptive field of 3 × 3 (the minimum size to capture the concepts of left/right, up/down, and center).

#### ResNet

From experience, the depth of the network is critical to the performance of the model. When the number of network layers is increased, the network can extract more complex feature patterns, so theoretically better results can be achieved when the model is deeper. However, when the network reaches a certain level, the depth of the network increases, and the accuracy of the network becomes saturated or even declined. Considering the ResNet (He et al., 2016), the author uses a residual block to avoid this situation.

#### Res2Net

Most of the existing methods use a hierarchical method to represent multi-scale features; nonetheless, Gao et al. (2021) have constructed hierarchical residual connections in a single residual block in a different way, proposing the Res2Net neural network building block. This module can express particle-level multiscale features and expand the range of receptive fields.

### Feature Expression Enhancement Module

#### Edge Attention Module

The enhancement effect of edge information in segmentation features has been verified in many studies (Zhang et al., 2019; Xing, 2020; Zhao et al., 2020; Zhou, 2020). The resolution of the low-level feature map was higher, and the edge information was richer. The network inputs the feature map obtained by the second- and low-level convolution to the edge attention module, extracts the edge information feature, and obtains the corresponding map. The difference between the generated edge map and edge truth map *G_e* corresponding to the true label is calculated using the binary cross entropy (BCE) loss function:


(1) $$L_{e\,d\,g\,e}=-\sum_{x=1}^{w}\sum_{\text{y}=1}^{h}\left[G_{e}\,l\,o\,g\,\left(S_{e}\right)+\left(1-G_{e}\right)\,l\,o\,g\,\left(1-S_{e}\right)\right]$$

where (*x*,*y*) represent the coordinates of the pixel points in the predicted edge map *S_e* and the edge truth map *G_e*. *G_e* is derived from the real label *G_s*. *w* and *h* represent the width and height of the feature map, respectively.

#### Parallel Partial Decoder Module

**FIGURE 5:**
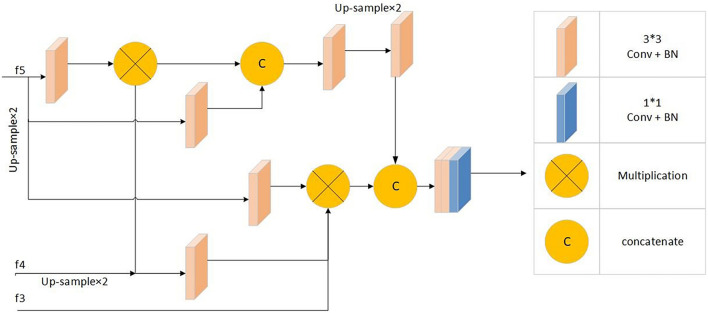
Parallel partial decoder module frame diagram: Including 1*1 convolution + batch normalization processing module, 3*3 convolution + batch normalization processing modules, multiplication operation and concatenate operation.

Segmentation through the combined use of high and low feature maps is a common method of medical segmentation (Qian et al., 2015; Zhou et al., 2018; Gu et al., 2019; Fan et al., 2020). However, because of their large size, low-level feature maps require more resources, and the effect on performance improvement is not obvious (Wu et al., 2019). The parallel partial decoder *Pd*(⋅) is used to fuse the high-level features to form a prediction map *S*_*g*_ = *Pd*(*f*_3_,*f*_4_,*f*_5_), guiding the input of the inverse attention module. Figure 5 shows parallel partial decoder module frame diagram.

#### Reverse Attention Module

Inspired by the study of Chen et al. (2018), the network uses the reverse attention module to extract richer information. The inverse attention module uses a progressive framework. Its information comes from the same convolutional layer, and it includes low-level features *f*_*2*_ and aggregated features from a higher level. This method can obtain more complex feature information and optimize the segmentation performance of the network.

The predicted feature map of the upper layer was expanded after the sigmoid activation, inversion, and smoothing. We multiplied the expanded feature by the high-level output feature (dot multiplication ⋅) and concatenated it with the edge attention feature *e*_*att*_(*f*_2_) to obtain the corresponding reverse attention feature output as demonstrated below:


(2) $$R_{i}=C\,\left(f_{i}\cdot A_{i},D\,o\,w\,\left(e_{a\,t\,t}\right)\right)$$

where *Dow*(⋅) and *C*(⋅) are the down-sampling concatenation operations, respectively.

**FIGURE 6:**
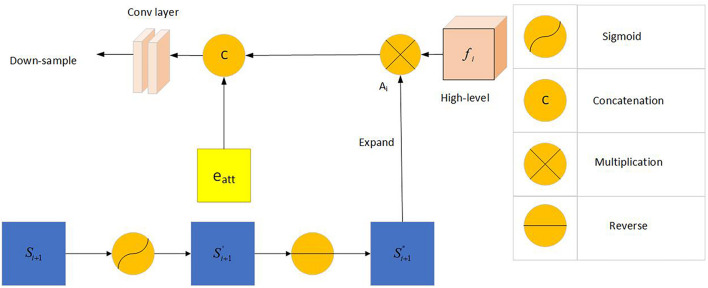
Frame diagram of the reverse attention module: Including Sigmoid activation function, concatenation operation, multiplication operation and reverse operation. *f_i* (i = 3,4,5) represents the feature information obtained by the picture through the *i*-th convolutional layer. *S*_*i* + 1_(i = 1,2) represents the information obtained by the combination of the reverse attention module information and the higher-level information. *A_i* represents the output after *S*_*i+1*_ expand.

Figure 6 shows frame diagram of the reverse attention module.

### Loss Function

The standard IoU loss and binary cross-entropy loss functions are the commonly used loss functions. The calculation formulas are as follows:


(3) $$L_{I\,o\,U}=1-\frac{\left|A\cap B\right|}{\left|A\cup B\right|}$$

(4) $$L_{B\,C\,E}=-\left(y\,l\,o\,g\,\left(p\right)+\left(1-y\right)\,l\,o\,g\,\left(1-p\right)\right)$$

(5) w⁢e⁢i⁢g⁢h⁢t=1+5⁢|A⁢V⁢G⁢(B)-B|

(6) $$L_{I\,o\,U}^{w}=1-\frac{\left(A\cap B\right)\cdot w\,e\,i\,g\,h\,t+1}{\left|A\cup B\right|\cdot w\,e\,i\,g\,h\,t-\left|A\cap B\right|\cdot w\,e\,i\,g\,h\,t+1}$$

(7) $$L_{B\,C\,E}^{w}=\frac{L_{B\,C\,E}\cdot w\,e\,i\,g\,h\,t}{w\,e\,i\,g\,h\,t}$$

where A represents the predicted map, B represents the true label, y is the true category, and p is the probability of the predicted category. *AVG*() is a function in the torch library, specifically torch.nn.AvgPool2d(kernel_size, stride = None, padding = 0), kernel size is the size of the window, stride is the stride of the window, and padding is implicit zero padding to be added on both sides.Input(*N,C,Hin,Win*), output(*N,C,Hout,Wout*), where


(8) $$H_{o\,u\,t}=\left\lfloor \frac{H_{i\,n}+2\times \text{padding}\,\left[0\right]-\text{kernel\_size}\,\left[0\right]}{\text{stride}\,\left[0\right]}+1\right\rfloor$$

(9) $$W_{o\,u\,t}=\left\lfloor \frac{W_{i\,n}+2\times \text{padding}\,\left[1\right]-\text{kernel\_size}\,\left[1\right]}{\text{stride}\,\left[1\right]}+1\right\rfloor$$

Where *N* stands for quantity, *C* stands for channel, *Hin* stands for input height, *Win* stands for input width, *Hout* stands for output height, and *Wout* stands for output width.

However, the weights assigned to each pixel by the above two loss functions are the same, and they do not focus on the extraction of complex pixel samples. This study combines the weighted IoU and weighted BCE loss functions to obtain a new loss function:


(10) $$L_{s\,e\,g}=L_{I\,o\,U}^{w}+L_{B\,C\,E}^{w}$$

To facilitate the calculation, each predicted feature map is restored to its original size through an up-sampling operation (for example, $S_{3}^{u\,p}$). Therefore, we rewrite the total loss function as:


(11) $$L_{t\,o\,t\,a\,l}=L_{s\,e\,g}\,\left(G_{s},S_{g}^{u\,p}\right)+L_{e\,d\,g\,e}+\sum_{i=3}^{5}L_{s\,e\,g}\,\left(G_{s},S_{i}^{u\,p}\right)$$

## Results

### Visualization of Segmentation Results

**FIGURE 7:**
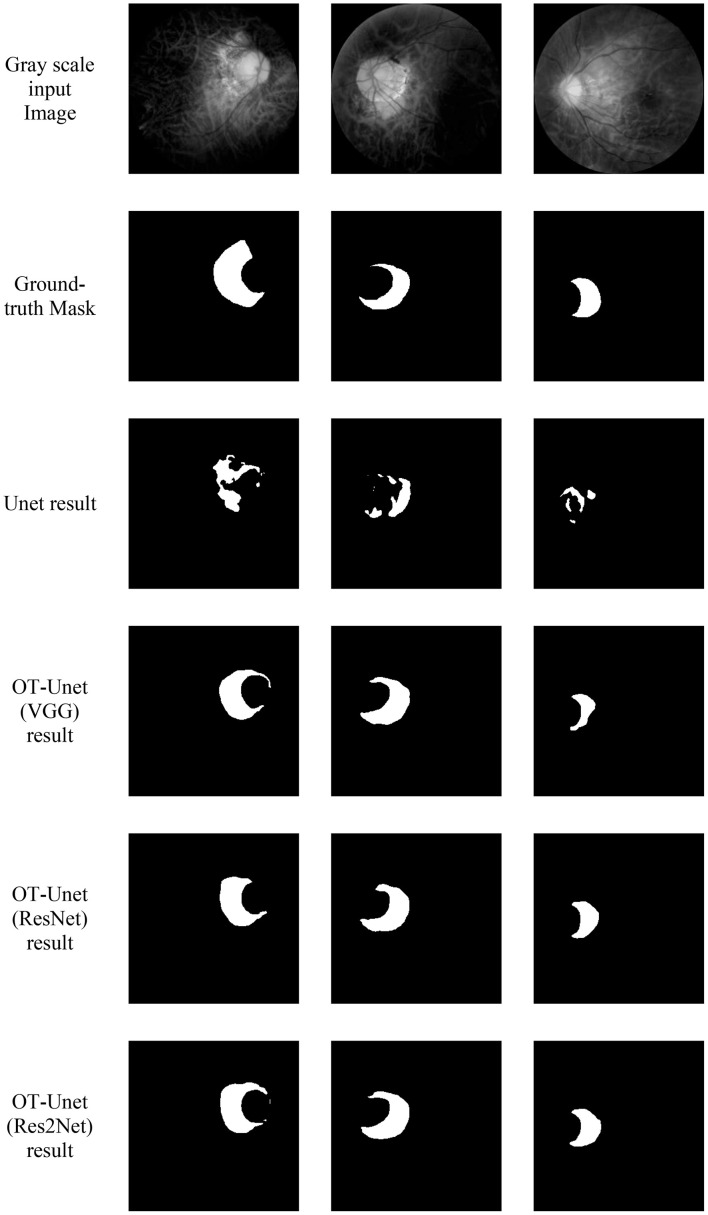
Visualized results of the segmentation of parapapillary atrophy in high myopia: It shows the Gray scale input Image, the Ground-truth Mask, and the segmentation results of Unet, OT-Unet (VGG), OT-Unet (ResNet), and OT-Unet (Res2Net).

Figure 7 shows the visualized results of high myopia grayscale images, real labels, and the visual segmentation results of the PPA. It can be observed that the OT-Unet segmentation algorithm has a better segmentation effect than the original Unet.

### Comparison of Segmentation Results

The following are the experimental segmentation results of the four networks.

**FIGURE 8:**
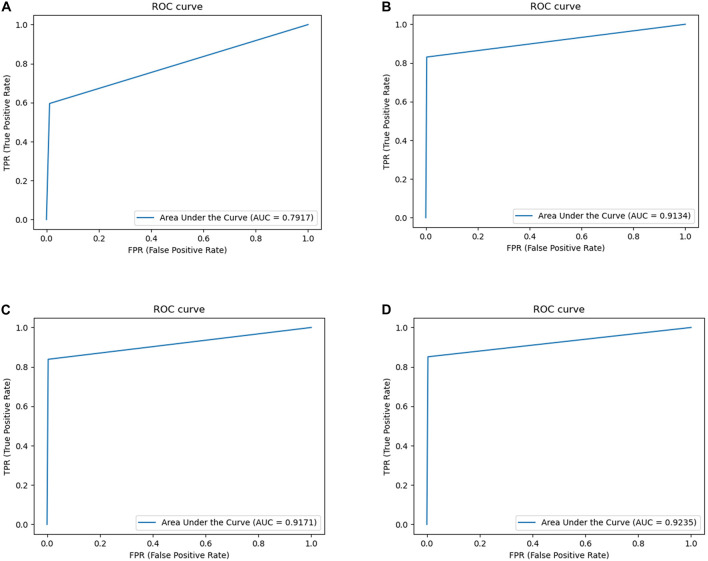
Experimental ROC curve diagram: **(A)** Unet (AUC = 0.7917); **(B)** VGG network (AUC = 0.9134); **(C)** ResNet (AUC = 0.9171); **(D)** Res2Net (AUC = 0.9235).

Considering Table 1, it can be observed that the segmentation network of this study has improved for all the indicators. It can be seen that OT-Unet is significantly better than Unet in all indicators. The three pre-training models of OT-Unet have different performance in various indicators. OT-Unet (VGG) has the best performance on the Precision and Specificity indicators; on the other indicators, OT-Unet (Res2Net) has the best performance. Figure 8 shows experimental ROC curve diagram of different segmented image networks.

**TABLE 1:** Comparison of segmentation results of different models.

Methods	Precision	Sensitivity	Specificity	AUC	IoU	DSC
Unet	0.7226	0.8303	0.9879	0.7917	0.4731	0.6413
OT-Unet (VGG)	**0.8227**	0.8225	**0.9963**	0.9134	0.7004	0.8086
OT-Unet (ResNet)	0.7980	0.8398	0.9957	0.9171	0.6877	0.8022
OT-Unet (Res2Net)	0.8020	**0.8450**	0.9958	**0.9235**	**0.7034**	**0.8101**

**DSC means Dice similarity coefficient.* Bold values indicate that the value is the largest in the same indicator.*

### Comparison of the Segmentation Results of Large and Small Lesions

Based on the relationship between the PPA and papilla diameter (PD), we divided the PPA that was less than or equal to one-third of the PD into small lesion areas (50 images), and the rest were large lesion areas (22 images). The best-performing Res2Net pre-training model was used for the segmentation to obtain the visualization and quantification results.

The quantification results of the segmentation of the large and small lesions are shown in Table 2. When OT-Unet segmented large lesions, its Precision, Sensitivity, Specificity and AUC scores were higher than those of small lesions. When segmenting a small lesion area, the lesion area is smaller than the optic disc and is more disturbed by it. Other non-lesion regions around the optic disc also interfere with the segmentation.

**TABLE 2:** Comparison of the segmentation results of OT-Unet (Res2Net) in different sizes of lesion areas.

Type of lesion	Precision	Sensitivity	Specificity	AUC	IoU	DSC
Small lesion	0.7479	0.8287	0.9956	0.9076	0.6517	0.7712
Large lesion	**0.9251**	**0.8820**	**0.9962**	**0.9352**	**0.8209**	**0.8986**

*Bold values indicate that the value is the largest in the same indicator.*

**FIGURE 9:**
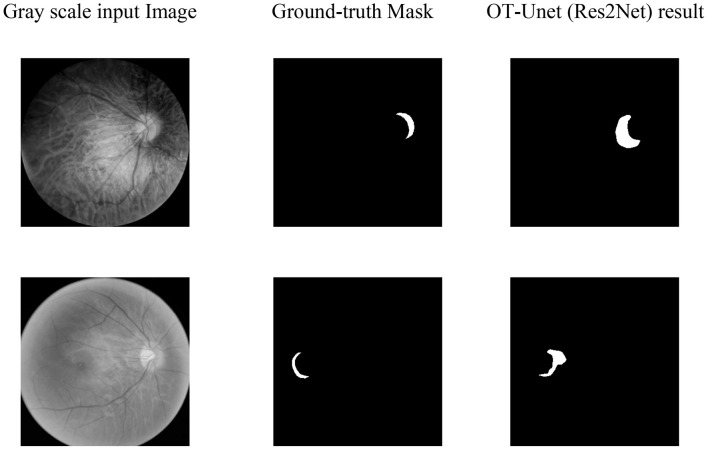
Segmentation of extremely small lesions: It can be seen that the segmentation prediction map does not correspond to the Ground-truth Mask at all.

According to the segmentation results, the segmentation performance of the OT-Unet on a large lesion area is better than that of a small lesion area in all indicators. When segmenting a small lesion area, the model is more susceptible to the influence of other areas around the optic disc, and even an extreme situation where the segmentation area does not match the real label at all occurs as shown in Figure 9.

## Discussion

As a method of auxiliary diagnosis, automatic segmentation of ophthalmic medical images can help ophthalmologists to understand a patient’s fundus more conveniently and clearly, indicating that this study is very valuable. The width of the PPA is positively correlated with the degree of myopia; therefore, early diagnosis is very important for patients with high myopia. Automatic segmentation of medical images can effectively extract and express image features with less preprocessing and reduce labor costs. Considering the introduction of many excellent segmentation network models and rapid improvement in image processor performance, deep learning can achieve higher segmentation accuracy.

Currently, there are few studies on the automatic segmentation of the PPA. The datasets on the Internet are very limited, manual labeling is time-consuming and laborious, and labeling accuracy cannot be guaranteed. In addition, the early PPA was crescent-shaped, which occurred near the optic disc with a small area. The difference between PPA and the brightness of the optic disc is not obvious, making it easy to be affected by the optic disc when splitting the PPA. Meanwhile, patients with high myopia often have a leopard-shaped fundus, which is a result of stretching the retina. Visible blood vessels affect the recognition and segmentation of the lesion area and reduce the accuracy of the segmentation. There is no obvious rule for the expansion of the PPA, and the shape and size of the PPA of the fundus in different patients are quite different. There was obvious pigmentation in the PPA, which will also affect the segmentation of PPA. It can be observed that various factors restrict the study of the PPA segmentation network.

The experimental results show that Unet can only segment the approximate outline of the lesion area, which is greatly affected by the optic disc. Also, the prediction map is irregular and has many noises. In the feature extraction stage, OT-Unet uses VGG, ResNet and Res2Net three pre-training models to extract richer feature information. Considering the problem of irregular contours of the lesion, this study adopts the method of adding the edge attention module to extract the contour features of the lesion area during the training phase of feature learning. The trained model can generate a clearer boundary prediction map. Solving the problem of the Unet being severely interfered by the optic disc, this study uses a parallel partial decoder and reverse attention modules to obtain more high-level and low-level fusion features. This helps the network learnt to distinguish between the PPA and optic disc, avoiding splitting the disc. OT-Unet also improved the loss function to get more accurate segmentation results.

However, according to the visualization results, the segmentation result still cannot completely avoid the interference of the optic disc, and the effect is not sufficient when segmenting the small-sized PPA. In the future, the learning ability of the low-level features of the network will be further enhanced to make the network perform better in the segmentation of small lesion areas.

Compared to the original segmentation network (Unet), the improved network has a better effect on lesion segmentation; however, there are still some areas to be improved. Only 288 training images are used in this study, which weakens the generalization ability of the network. Considering high myopia, the size and shape of the PPA at different stages of development are very different, and the use of the same segmentation strategy will reduce the segmentation effect. The improved network makes it difficult to segment small-sized PPA. In the future, it will be necessary to use larger datasets and use data enhancement methods for expansion. Before segmentation, a classification network can be used to classify the PPA according to early, middle, and late stages. Subsequently, based on the characteristics of the para-optical atrophy in the different stages, targeted segmentation strategies can be formulated for segmentation. Regarding the loss function, this study does not systematically study the weight distribution of the weighted IoU and binary cross-entropy loss functions; nonetheless, it simply adds them. In the future, the relationship between these two loss functions can be explored, and a more appropriate weight distribution of the loss functions can be found to improve the segmentation performance of the network.

## Conclusion

Considering the segmentation task of PPA for high myopia, this study proposes an OT-Unet algorithm network. In this study, three pre-training models (VGG, ResNet, and Res2Net) are used in the Unet for the extraction of convolutional features. Between the high- and low-level convolutions, this study introduces an edge attention module to extract edge feature maps and enrich the network information. Multi-scale high-level feature maps use a parallel partial decoder module to perform feature fusion and obtain global information. The network also uses a reverse attention module which uses a progressive framework to extract high- and low-level feature information. Considering the loss function, the network combines the weighted IoU and binary cross-entropy loss functions to increase the weight of the complex pixels. This shows that the improvement of the network structure and loss function significantly improves the segmentation performance of the network and obtains a better segmentation effect than the Unet in the segmentation of the lesion area. Compared to the Unet, the improved OT-Unet is superior for all the evaluation criteria.

## Data Availability Statement

The raw data supporting the conclusions of this article will be made available by the authors, without undue reservation.

## Author Contributions

CW contributed to the conception of the study and performed the experiments. JW performed the data analyses and wrote the manuscript. HL helped to perform the analysis with constructive discussions. ZY, CHW, and QJ collected the data and directed the writing of the manuscript. GC contributed significantly to analysis and manuscript preparation. YX revised the manuscript. WY supervised the whole study. All authors contributed to the study and approved the submitted version.

## Conflict of Interest

The authors declare that the research was conducted in the absence of any commercial or financial relationships that could be construed as a potential conflict of interest.

## Publisher’s Note

All claims expressed in this article are solely those of the authors and do not necessarily represent those of their affiliated organizations, or those of the publisher, the editors and the reviewers. Any product that may be evaluated in this article, or claim that may be made by its manufacturer, is not guaranteed or endorsed by the publisher.
